# Novel Calcium-Sensing Receptor (*CASR*) Mutation in a Family with Autosomal Dominant Hypocalcemia Type 1 (ADH1): Genetic Study over Three Generations and Clinical Characteristics

**DOI:** 10.1159/000529833

**Published:** 2023-02-22

**Authors:** Amnon Zung, Galia Barash, Ehud Banne, Michael A. Levine

**Affiliations:** a Division of Pediatrics and Pediatric Endocrinology Unit, Kaplan Medical Center, Rehovot, Israel;; b The Hebrew University of Jerusalem, Jerusalem, Israel;; c Pediatric Endocrinology Unit, Shamir (Assaf Harofeh) Medical Center, Tzrifin, Israel;; d The Genetic Institute, Edith Wolfson Medical Center, Holon, Israel;; e Center for Bone Health and Division of Endocrinology and Diabetes, Children’s Hospital of Philadelphia and University of Pennsylvania Perelman School of Medicine, Philadelphia, PA, USA

**Keywords:** Calcium-sensing receptor, Autosomal dominant hypocalcemia, Hypercalciuria

## Abstract

**Introduction:**

Activating mutation of the calcium-sensing receptor gene (*CASR*) reduces parathyroid hormone secretion and renal tubular reabsorption of calcium, defined as autosomal dominant hypocalcemia type 1 (ADH1). Patients with ADH1 may present with hypocalcemia-induced seizures. Calcitriol and calcium supplementation in symptomatic patients may exacerbate hypercalciuria, leading to nephrocalcinosis, nephrolithiasis, and compromised renal function.

**Methods:**

We report on a family with seven members over three generations with ADH1 due to a novel heterozygous mutation in exon 4 of *CASR*: c.416T>C.

**Results:**

This mutation leads to substitution of isoleucine with threonine in the ligand-binding domain of CASR. HEK293T cells transfected with wild type or mutant cDNAs demonstrated that p.Ile139Thr substitution led to increased sensitivity of the CASR to activation by extracellular calcium relative to the wild-type CASR (EC_50_ of 0.88 ± 0.02 mM vs. 1.1 ± 0.23 mM, respectively, *p* < 0.005). Clinical characteristics included seizures (2 patients), nephrocalcinosis and nephrolithiasis (3 patients), and early lens opacity (2 patients). In 3 of the patients, serum calcium and urinary calcium-to-creatinine ratio levels obtained simultaneously over 49 patient-years were highly correlated. Using the age-specific maximal-normal levels of calcium-to-creatinine ratio in the correlation equation, we obtained age-adjusted serum calcium levels that are high enough to reduce hypocalcemia-induced seizures and low enough to reduce hypercalciuria.

**Conclusion:**

We report on a novel *CASR* mutation in a three-generation kindred. Comprehensive clinical data enabled us to suggest age-specific upper limit of serum calcium levels, considering the association between serum calcium and renal calcium excretion.

## Introduction

The calcium-sensing receptor (CASR) serves as the main calcium sensor in maintaining extracellular calcium homeostasis. The human CASR is a G-protein-coupled receptor with a 612-amino acid extracellular domain, a transmembrane region composed of 7 transmembrane helices, and a 216-amino acid C-terminal intracellular region. The receptor is encoded by the human *CASR* gene located on chromosome 3q21.1 [1]. It is ubiquitously expressed in mammalian tissues, but its expression in the parathyroid gland and ascending limb of the loop of Henle in the renal tubules is most important to its role in calcium homeostasis. Elevation in serum calcium induces a cascade that starts with Gq/11-protein-dependent stimulation of phospholipase C activity, causing accumulation of inositol 1,4,5-trisphosphate and a rapid increase of cytosolic Ca^2+^ concentration, which eventually leads to a decrease in parathyroid hormone (PTH) secretion and a reduction in renal tubular reabsorption of calcium [2]. This cascade enables both the parathyroid gland and the renal tubules to respond to fluctuations in extracellular calcium concentration in order to restore its steady state.

Activating mutations in *CASR* increase the receptor’s sensitivity to extracellular ionized calcium, leading to decreased synthesis and secretion of PTH from the parathyroid gland and increased renal excretion of calcium, independently of the action of PTH. Activating mutations in *CASR* cause autosomal dominant hypocalcemia type 1 (ADH1), alternatively termed familial hypercalciuric hypocalcemia [3, 4]. Patients with ADH1 present with hypocalcemia, hypomagnesemia, hyperphosphatemia, hypercalciuria, and inappropriately low or normal levels of PTH. Clinically, many ADH1 patients are asymptomatic. However, symptomatic patients present with neuromuscular symptoms such as paresthesia, muscle cramping, carpopedal spasm, and seizures. Treatment with activated vitamin D analogs and calcium supplementation should be reserved for symptomatic patients, with a goal calcium level that is near the lower limit of an age-dependent reference range to avoid excessive urinary calcium excretion, and yet high enough to alleviate symptoms [5]. This approach aims to reduce hypercalciuria-induced nephrocalcinosis and nephrolithiasis, with subsequent compromised renal function [3, 4]. A total of 334 different variants of *CASR* (activating and inactivating mutations, combined) have been reported, 10 and 245 of them considered likely pathogenic and pathogenic, respectively (http://databases.lovd.nl/shared/genes/CASR).

In the present study, we describe a three-generation kindred with ADH1 caused by a novel activating mutation of *CASR*. Long-term clinical data gathered from the medical records of some of the patients enabled us to suggest upper limit of serum calcium levels associated with normal-range renal calcium excretion in ADH1.

## Materials and Methods

### Subjects

The proband was born in the 39th week of gestation by caesarean section to a mother with hypocalcemia. The mother was treated with calcium and magnesium supplementation throughout the pregnancy. His birth weight was 3,240 g and 1- and 5-min Apgar scores were 9 and 10, respectively. Shortly after birth, the mother’s serum calcium level was 6.4 mg/dL (normal range 8.8–10.8 mg/dL). At the same time, the proband had a calcium level of 7.7 mg/dL (normal range 8.8–10.8 mg/dL), phosphate level of 6.9 mg/dL (normal range 3.3–5.4 mg/dL), and PTH level below the assay’s limit of detection. Oral supplementation of calcium glubionate and alfacalcidol was then initiated based on a postulated diagnosis of hypoparathyroidism. On the fourth day of life, he presented with a generalized tonic-clonic seizure that lasted for 3 min, followed by two additional short-term seizures. At that time, his serum calcium level was 6.0 mg/dL (ionized calcium 0.83), phosphate was 8.6 mg/dL, and magnesium was 1.92 mg/dL (normal range 1.7–2.1 mg/dL). The proband was transferred to the neonatal intensive care unit where he was initially treated with intravenous calcium gluconate, followed by oral supplementation of calcium glubionate and alfacalcidol until a serum calcium level of 8.2 mg/dL was reached.

Following this case, 10 other family members over three generations were evaluated, 6 of them with hypocalcemia. Clinical and biochemical data were obtained retrospectively from the medical charts of the 7 patients with hypocalcemia. All 11 subjects underwent genetic evaluation following written informed consent. Written informed consent was also obtained from the participants (or their legal guardian in the case of minors) to publish their case.

### Analysis of CASR

We performed Sanger sequencing of the *CASR* gene (NM_001178065.1) in the proband, including the exons and their boundaries, without the promotor. In other members of the family, only exon 4 of *CASR* was sequenced, based on the results in the proband. We also used PCR-based site-directed mutagenesis to insert the p.Ile139Thr substitution into a wild-type human CASR cDNA. We transfected HEK293T cells grown on collagen-coated coverslips with wild type or mutant cDNAs to that included a GFP sequence in the N-terminus. We then examined the expression of recombinant CASR proteins by confocal microscopy [6]. We also transfected HEK293T cells with wild type or mutant cDNAs and determined sensitivity to extracellular calcium after loading cells with Fluo-4 tetra(acetoxymethyl)-ester and Fura-red AM red as previously described [7]. HEK293T cells were transfected with either wild type or mutant CaSR cDNAs in six-well plates and after 48 h the cells were detached, resuspended in Hanks’ balanced salt solution (HBSS) and simultaneously loaded with Fluo-4 tetra(acetoxymethyl)-ester (AM; 2 μM) and Fura-red AM (2 μM) for 45 min at room temperature. Cells were washed in HBSS containing calcium chloride (0.1 mM). Aliquots (50 μL) of suspended cells were mixed with an equal volume of HBSS containing calcium chloride to yield final calcium concentrations of 0.1, 0.3, 0.6, 1, 1.8, 3.2, 5.6, or 10 mM. Immediately after mixing, each cell sample was analyzed on an Accuri C6 flow cytometer (BD Biosciences). The ratio between fluorescence intensities recorded in the FL-1 channel (530/30 nm; Fluo-4 AM) and the FL-3 channel (670 nm long pass; Fura-red AM) was calculated for each cell as a derived parameter using FlowJo 7.6.5 (Tree Star Inc). Curves were fitted to data using the four-parameter Hill equation in GraphPad Prism. EC50 values were determined for each cell type tested, and the mean EC50 values were calculated from data obtained from four sets of measurements as described [7].

## Results

### Sequencing of CASR

**Fig. 1.**
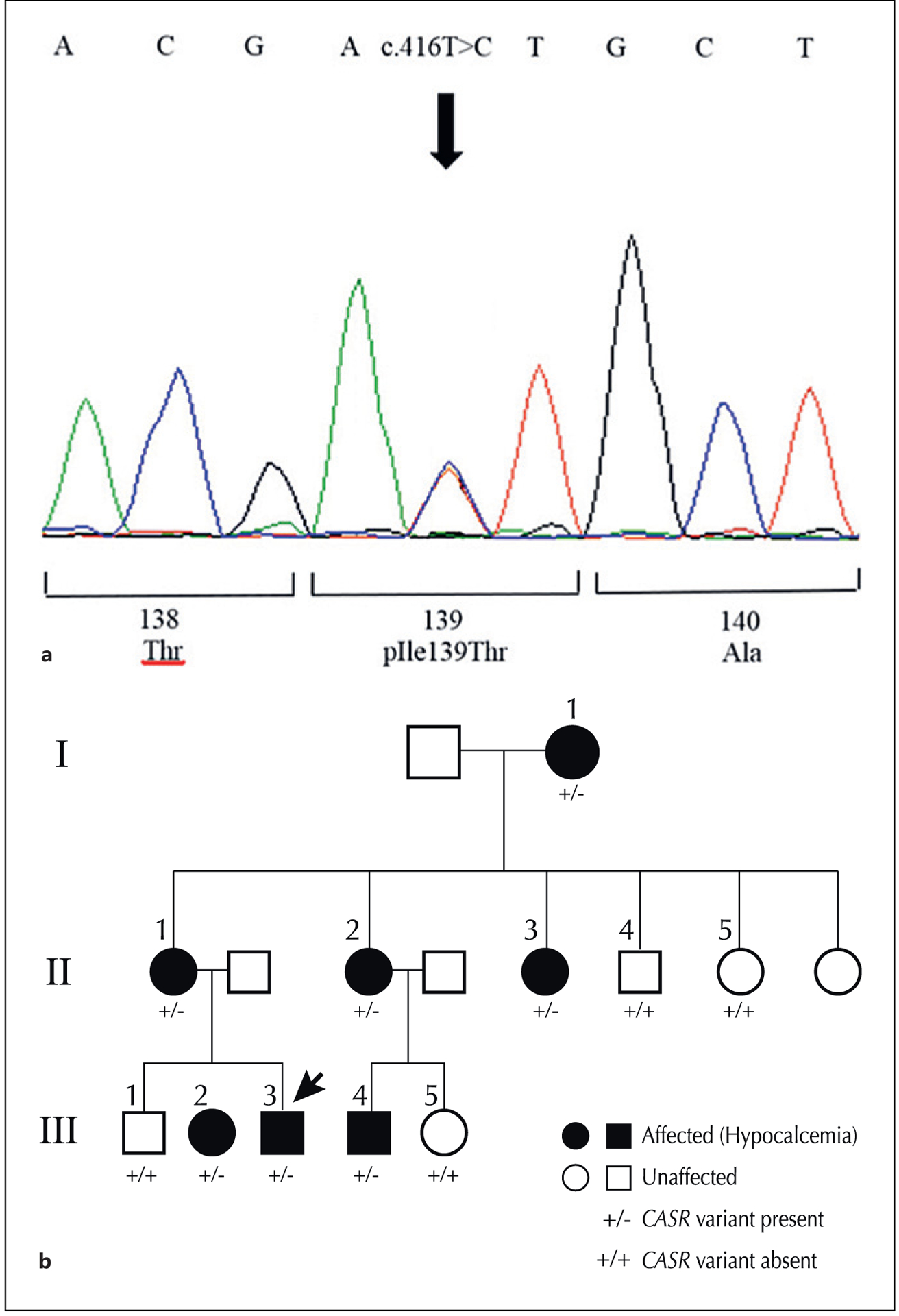
**a** The heterozygous T>C transition at nucleotide c.416 in exon 4 of *CASR* gene was identified in the affected members of the family, which changed ATT codon to ACT and is predicted to result in a substitution of isoleucine with threonine in the ligand-binding domain of CASR. **b** Pedigree of the family with ADH1. Squares represent male and circles represent female family members. Affected and unaffected individuals are represented by filled and open symbols, respectively. The index case is marked by an arrow.

Sanger sequencing of the entire gene in the proband revealed a novel heterozygous mutation in exon 4 of *CASR*: c.416T>C (Fig. 1a). We analyzed the significance of the mutation using the VarSome website [8], according to the American College of Medical Genetics and Genomics (ACMG) guidelines [9]. We used the ClinVar database [10] to gather more information on the *CASR* gene. For comparison with the normal population, we verified the Genome Aggregation Database (gnomAD) [11].

The mutation was reported in the CASR database as a variant of unknown significance (http://www.casrdb.mcgill.ca). In the ClinVar database [10], it was reported as a change of uncertain significance, and on the VarSome website [8], it was analyzed by the ACMG criteria as likely pathogenic [9]. It is a rare variant that was not reported in gnomAD [11]. This mutation leads to substitution of isoleucine with threonine (p.Ile139Thr; g.121976158; g.121976158) at the cysteine-rich domain of the extra-cellular domain of CASR.

**Fig. 2.**
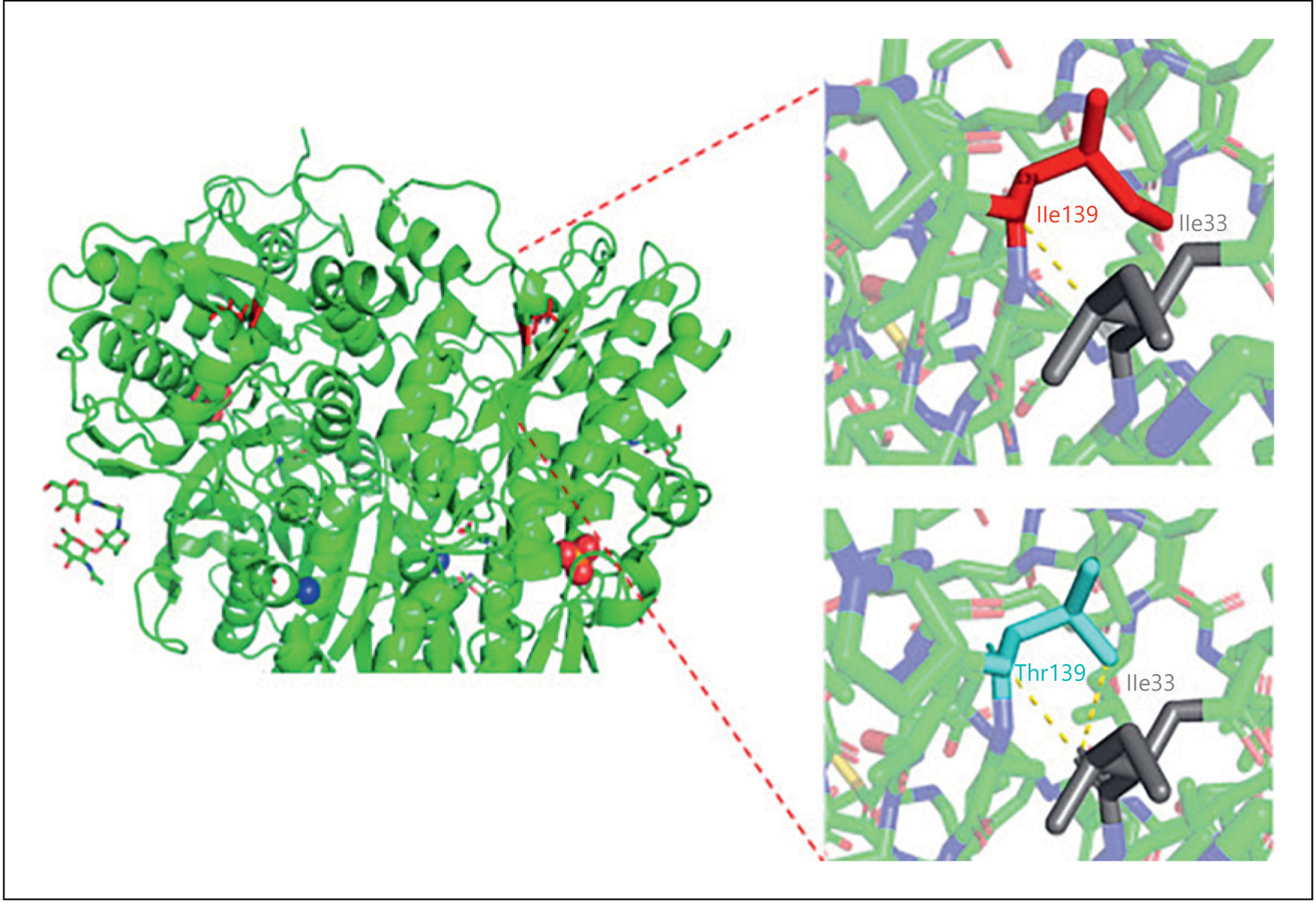
Ribbon diagram of the CASR structure (left) and localization of the mutation within the ligand-binding domain of the receptor in the extracellular domain (position 139 in red). Modeling of the wild-type Ile139 (Upper right) and mutant Thr139 residue (Lower right) using an inactive state CASR structure obtained from high-resolution cryo-EM and Pymol showed the side chain changing from large aliphatic residue to a small neutral one. The introduction of a mutant Thr139 residue (pale blue) is predicted to form an additional hydrogen bond between residue Thr139 and Ile33 (dashed yellow lines).

**Fig. 3.**
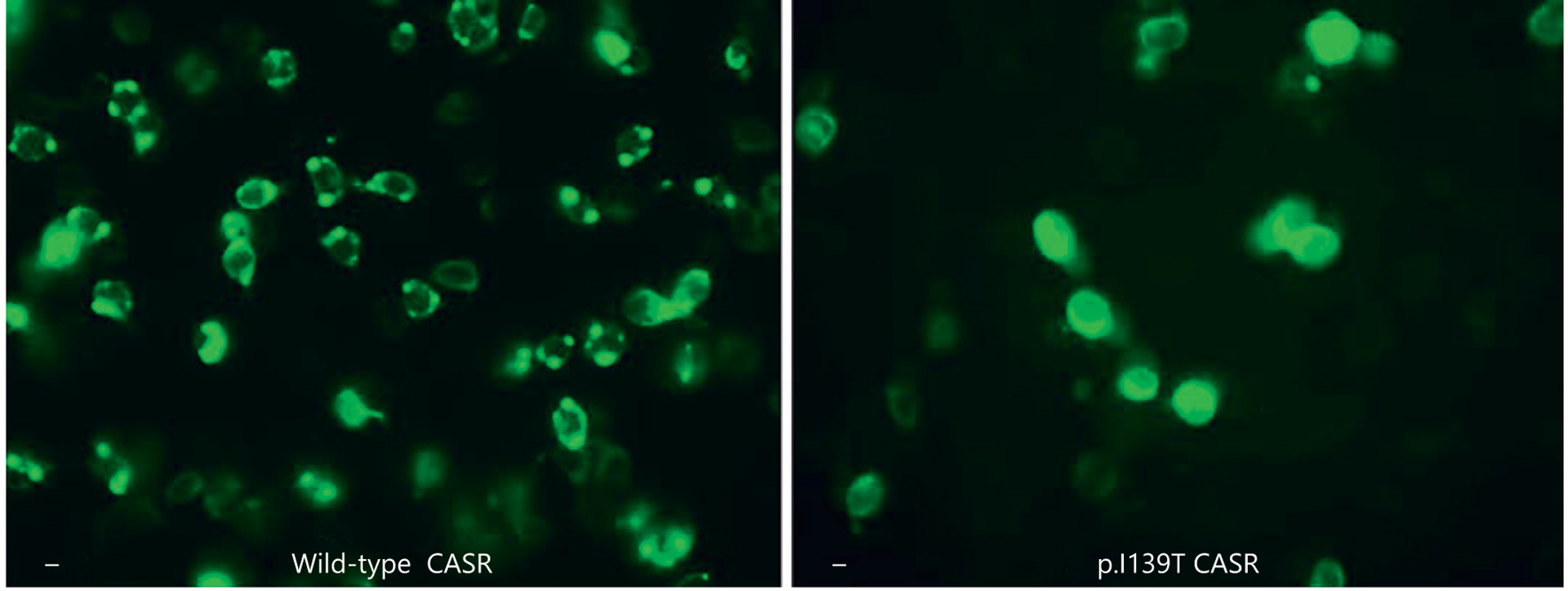
Cellular expression of wild-type and mutant CaSRs in transfected HEK293T cells. Confocal fluorescence microscopy was performed on cells that had been transiently transfected with chimeric CaSR-EGFP cDNAs that encode wild-type (left panel) or mutant (right panel, p.Ile139Thr) CaSR-green fluorescent protein fusion proteins. Both CaSR isoforms were similarly expressed at the periphery of the HEK293T cells, consistent with cell surface localization of the p.Ile139Thr CASR. Scale bar is 10 microns.

**Fig. 4.**
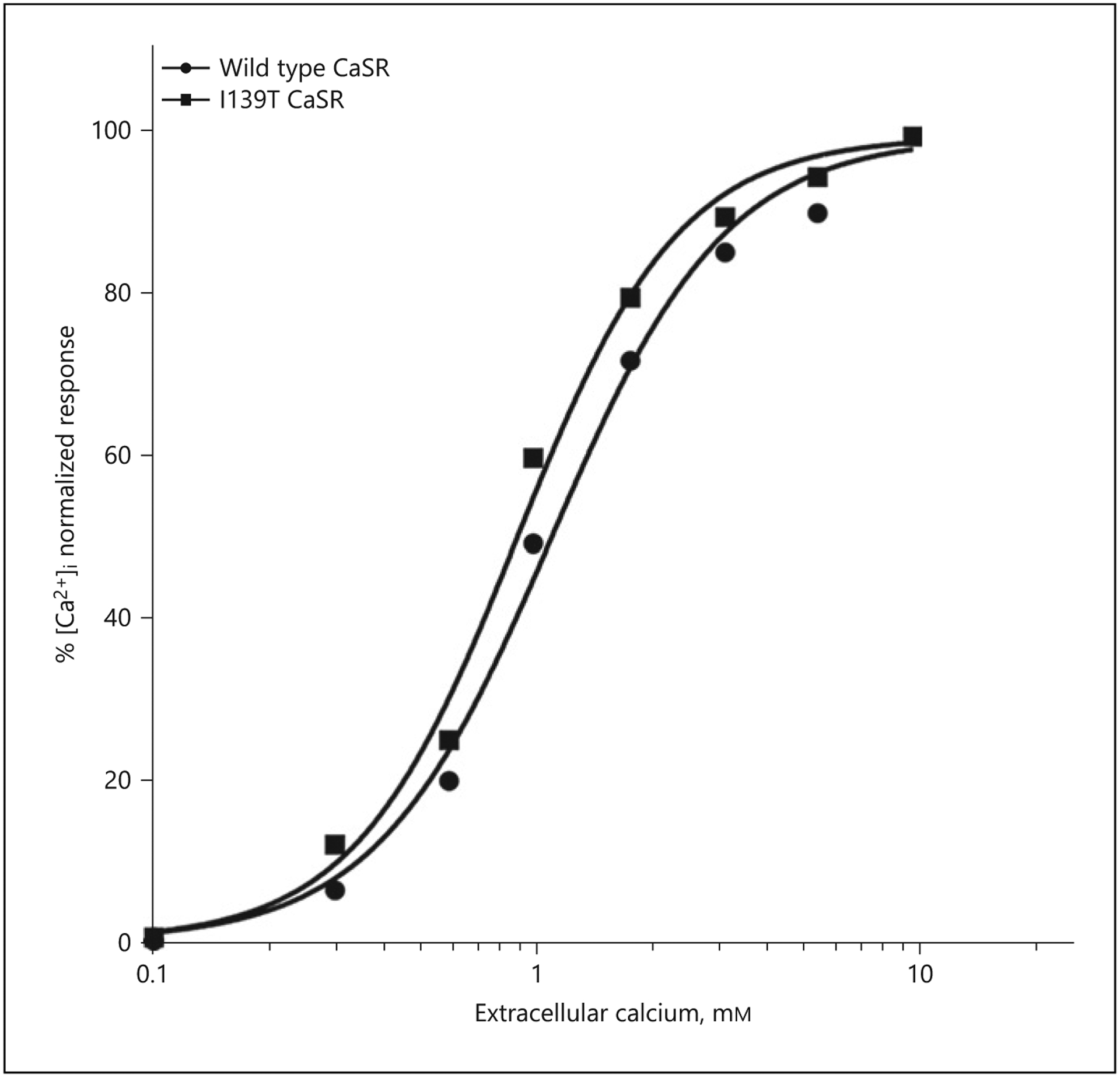
Intracellular Ca^2+^ responses to changes in extracellular Ca^2+^ concentration in HEK293T cells expressing the wild-type or mutant CaSRs. The dose-response curve of the I139T mutant showed a leftward shift relative to that of the wild-type receptor. The curves shown are representative of four independent experiments.

We modeled the wild-type Ile139 and mutant Thr139 residues using an inactive state CASR structure obtained from high-resolution cryo-EM (PDB: 7M3G) [12] and Pymol [13]. As shown on Figure 2, the side chain changing from large aliphatic residue to a small neutral one was predicted to form an additional hydrogen bond between residue Thr139 and Ile33 (analyzed by the webserver Arpeggio; https://biosig.lab.uq.edu.au/arpeggioweb). Confocal images showed that wild type and p.Ile139Thr CASRs were similarly expressed at the periphery of the HEK293T cells, consistent with cell surface localization of the p.Ile139Thr CASR (Fig. 3). After transfection of cells with wildtype or mutant CASR plasmids, single-cell fluorescence measurements of intracellular calcium (Ca^2+^) were obtained from multiple cells. These studies demonstrated that the p.Ile139Thr substitution led to a significant increase (*p* < 0.005 by two-tailed Student’s *t* test, four independent experiments) in sensitivity of the CASR to activation by extracellular calcium (EC_50_ of 0.88 ± 0.02 mM, mean ± SD) relative to the wild-type CASR (EC_50_ of 1.1 ± 0.23 mM) (Fig. 4).

The c.416T>C mutation cosegregated with hypocalcemia in all seven individuals, whereas four unaffected normocalcemic members of the family did not exhibit the mutation (Fig. 1b). Patient III-5 was diagnosed in intrauterine screening.

### Clinical and Biochemical Studies

The clinical and biochemical data of the seven affected members of the family are described in Table 1. Two members presented with seizures during the neonatal period: patient III-3 (the proband) had three episodes of generalized tonic-clonic seizures and patient III-4 had two seizure episodes at 12 days and 7 months of age (both episodes occurred during febrile disease). The corresponding serum calcium levels at the time of the first convulsion in these patients were 6.0 mg/dL (ionized calcium 0.83 mg/dL) and 7.1 mg/dL, respectively.

Three patients had nephrocalcinosis, and two of these also had nephrolithiasis (Table 1). Two of the patients (I-1 and II-1 in Table 1) presented with impaired renal function (serum creatinine >1.2 mg/dL) and estimated glomerular filtration rate (eGFR) <60 mL/min/1.73 m^2^ at their last assessment at 50 and 29.3 years of age, respectively. eGFR was calculated using CKD-EPI formula [14]. In addition, two of the family members developed lens involvement: patient I-1 developed early cataract at 27 years of age and patient II-2 developed focal opacity in the posterior capsule of the lens at 2.3 years of age. All patients with nephrocalcinosis, nephrolithiasis and early appearance of lens opacity were treated with calcitriol and calcium supplementation at various doses.

### Calculation of Optimal Age-Dependent Serum Calcium Levels

**Fig. 5.**
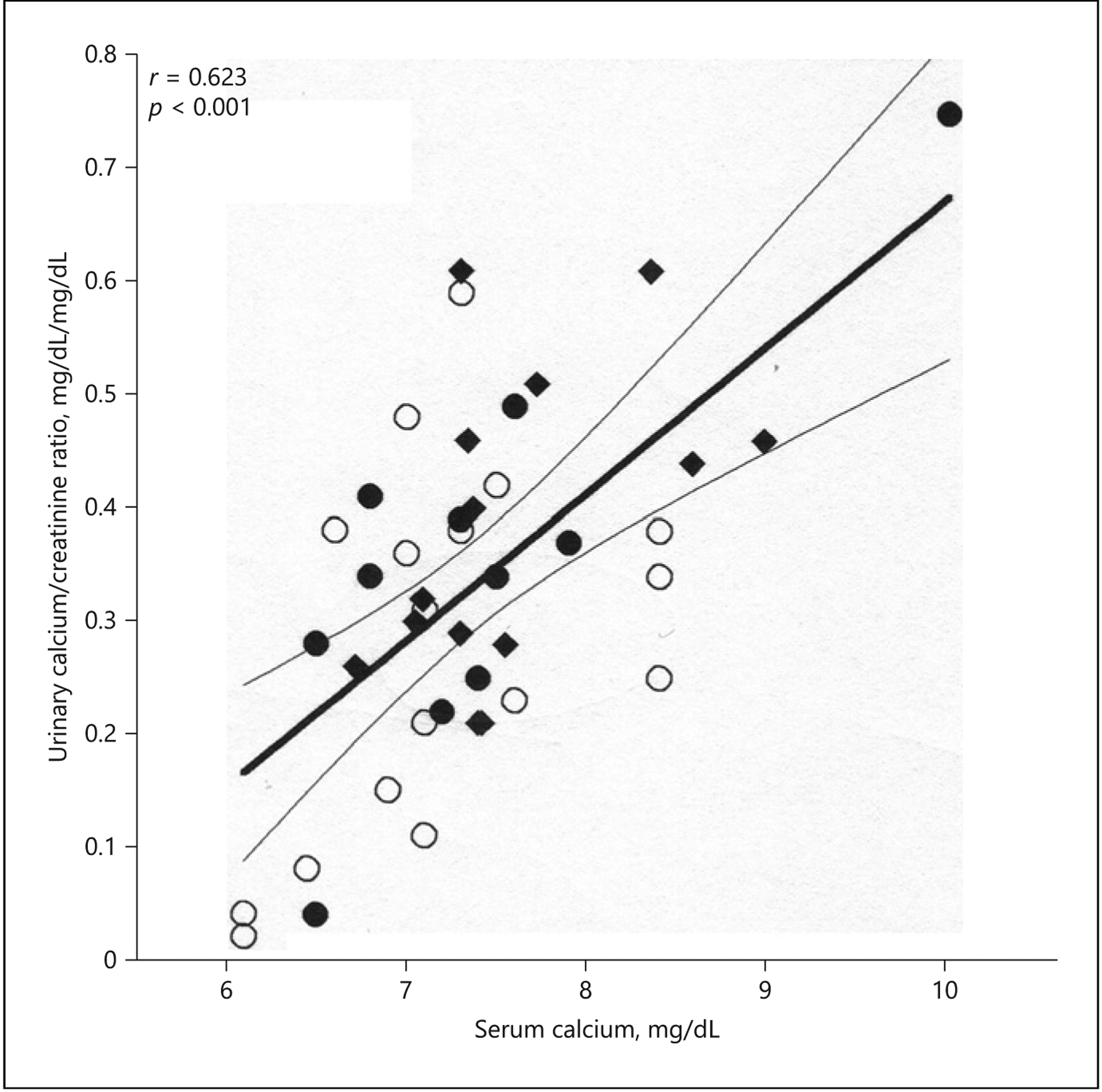
Correlation between serum calcium and urinary calcium-to-creatinine ratio in three members with ADH1: II-2 (open circles), II-3 (filled circles), and III-2 (diamonds) (Table 1; Fig. 1b). Data are presented as regression line with 95th confidence interval.

For 3 patients (II-2, II-3, III-2) with long-term follow-ups, we obtained serum calcium levels and urinary calcium-to-creatinine (Ca/Cr) ratios (from spot urine samples) that were measured simultaneously over several years from infancy to early adulthood (*n* = 42 paired measurements). 7 of the measurements were obtained during the first 3 years of life, 30 measurements during childhood (3.1–12 years), 3 measurements during adolescence (12.1–18 years), and 2 measurements during adulthood (>18 years). These measurements were positively correlated: serum calcium = 6.40 + 2.96(Ca/Cr ratio); *R* = 0.623, *p* < 0.001 (Fig. 5). The relationship of serum calcium with urinary Ca/Cr ratio was not affected by patients’ age or renal function expressed by serum creatinine and eGFR (calculated by stepwise linear regression). Furthermore, neither serum calcium levels nor urinary Ca/Cr ratios were correlated with eGFR. To calculate the highest serum calcium level associated with calcium excretion in the upper normal range, we plugged age-adjusted upper levels of urinary Ca/Cr ratios [15, 16] into the correlation equation. The two reports that described the upper urinary Ca/Cr levels (95th percentile) included healthy subjects from the USA (15) and Switzerland (16) that were mainly Caucasians, similar to the population in Israel. The European study (16) was much larger and contained more age-groups by comparison to the American study; therefore, we based our serum calcium calculations on age-ranges reported in this study (from birth to 7 years of age). Notably, only the American study contained data on adult subjects. From 7 years of age onward, we combined the data on the upper levels of urinary Ca/Cr ratios of both studies, since these levels were in a narrow range of 0.22 to 0.25, which yielded a similar calculated maximal serum calcium value of 7.1 mg/dL. The upper limit levels of serum calcium are given in Table 2.

## Discussion

We report a novel heterozygous mutation (c.416T>C) in *CASR* that caused ADH1 in all seven family members with hypocalcemia. This mutation leads to a substitution of isoleucine with threonine, with the formation of an additional hydrogen bond that occurs between the backbone of Ile33 and the residue side chain of Thr139. The replacement of the nonpolar amino acid isoleucine with the polar amino acid threonine, with the additional hydrogen bond, may affect the hydrophobic packing of the protein core.

In patients with ADH1, two mechanisms contribute to hypercalciuria: first, low concentrations of PTH, which normally induces renal reabsorption of calcium from the primary filtrate; second, increased activation of the mutated CASR through extracellular calcium in the thick ascending limb of the Loop of Henle, which leads to even more profound hypercalciuria for any given blood calcium level [5]. In addition, calcium and calcitriol administration, which is aimed at alleviating hypocalcemia in symptomatic patients, may further exacerbate hypercalciuria, which may subsequently induce nephrocalcinosis, nephrolithiasis, and impaired renal function in patients with ADH1 [17]. It has been estimated that 10% of ADH1 patients develop nephrocalcinosis and nephrolithiasis in association with hypercalciuria [18]. In a study by Pearce and colleagues [3], 8 out of 9 patients with hypercalciuria developed renal calcification, and seven of them developed renal impairment. Renal calcification and subsequent renal impairment were also developed in 7 other patients during vitamin D therapy [3]. These observations were confirmed in another study where all 8 patients with hypercalciuria developed nephrocalcinosis [19]. Therefore, it has been suggested that treatment should be reserved only for symptomatic patients, with the lowest possible goal calcium levels to alleviate symptoms [3, 5, 18–21]. In practice, this guideline is difficult to implement, and specific serum calcium levels are rarely recommended for patients with ADH1 [18, 19]. Lienhardt et al. [19] suggested that serum calcium levels be kept above 7.8 mg/dL irrespective of age because none of the patients with severe clinical signs of hypocalcemia had serum calcium values above this threshold. Sorheim et al. [22] stated that ADH1 patients should maintain serum calcium in the range of 7.6–8.4 mg/dL. In addition, urinary calcium excretion should be carefully monitored to minimize nephrocalcinosis and nephrolithiasis [23]. Unlike these studies that suggested fixed ranges of serum calcium, we aimed to define in our study the highest serum calcium levels that will not jeopardize renal function, while keeping it in the lowest possible level to avoid neurological symptoms. We first performed a correlation analysis between serum calcium levels and urinary Ca/Cr ratio, a recognized index of renal calcium excretion. We then used the highest normal levels of urinary Ca/Cr ratio (95th percentile) [15, 16] in the resulting correlation equation and came up with the highest serum calcium levels that correlate with renal calcium excretion in the upper normal range. Since urinary calcium decreases with age [15, 16, 24], the upper limit levels of calcium ranged from 8.8 mg/dL in the first year of life to 7.1 mg/dL in late childhood and adulthood. In previous reports, serum calcium levels that were associated with seizures were below our recommended age-related levels of calcium [4, 19, 20, 25, 26]. In a literature review of 16 papers that included 48 children with ADH1, 24 of the 25 children with seizures had serum calcium levels below our recommended age-related levels, with a mean calcium level of 6.3 mg/dL [21]. Therefore, our age-adjusted levels of serum calcium are low enough to prevent hypercalciuria and high enough to prevent seizures. Our correlation equation was quite similar to the one that we calculated from raw data of 26 measurements (in 20 patients) in Pearce et al. [3]: serum calcium = 6.65 + 3.12(Ca/Cr) (*R* = 0.651; *p* < 0.001). Notably, while measurements in the latter study were mainly obtained during adulthood, the measurements in the present study covered a wide range of ages. Our approach, however, might not guarantee prevention of renal impairment in all cases. Although most patients with hypercalciuria in Pearce et al. [3] developed some degree of renal impairment, 3 out of 7 patients that received vitamin D treatment developed renal calcification and renal impairment despite urinary Ca/Cr being in the high-normal range. Our approach contradicts the conclusion drawn by Lienhardt et al. [19] that serum calcium level is not a good predictor of hypercalciuria or nephrocalcinosis because both conditions can develop when serum calcium remains below the normal range. Unlike that study, we suggest age-adjusted levels of serum calcium, where different levels of hypocalcemia should be maintained at different ages to prevent hypercalciuria (Table 2). The importance of minimizing hypercalciuria is demonstrated in our study where 3 out of 7 patients with ADH1 had nephrocalcinosis, alone or with nephrolithiasis.

We found lens involvement at a relatively early age in two of the family members. Early presentation of cataracts has been previously reported as congenital cataracts [27] or as cataracts presenting in the fifth decade of life [28]. Cataracts have also been reported as part of various ectopic calcifications in a mouse model of activating *CASR* mutation [29].

There are several limitations to this study. First, the equation we suggested for the correlation between serum calcium and urinary Ca/Cr ratio was based on 3 patients only and 42 serum and urine paired values. Furthermore, urinary Ca/Cr values were taken from two studies that included mainly Caucasian population. Therefore, the equation should be evaluated in larger groups of ADH1 patients with ethnic diversity in order to assure its validity in various populations.

Second, there is a phenotypic variation in ADH1, even among family members with the same genotype [22]. Therefore, we cannot assure that the equation we suggest is applied for all other ADH1 patients with various mutations in the *CASR* gene, which might maintain different correlation between serum calcium and urinary Ca/Cr ratio. Nonetheless, when we calculated the correlation of these parameters in previously reported 20 ADH1 patients with five different mutations in the *CASR* gene [3], we came up with an equation that is almost identical to the equation in our cohort.

Finally, we used spot urinary calcium to creatinine ratio for the correlation analysis rather than the 24 h urinary calcium excretion. 24 h urine collection is considered the gold standard for evaluating calcium excretion and can vary from urine spot sample. Since many of the measurements obtained during infancy and early childhood, 24 h urine collection was impractical in these cases. For older patients, we recommend to rely on 24 h urine collection in order to evaluate urinary excretion of calcium.

In conclusion, we describe a novel mutation in *CASR* in a family with ADH1. Data on serum calcium levels and urinary calcium secretion obtained in 3 of the patients over 49 patient-years enabled us to recommend age-dependent serum calcium levels. In line with the current recommendations, we suggest to keep serum calcium levels high enough to alleviate symptoms, while considering our age-adjusted levels as upper limits in asymptomatic patients. Practically, serum calcium levels should optimally be kept below the suggested upper limits, as long as the patient is free from hypocalcemic symptoms. Adjustments to these levels may be required if hypocalcemic symptoms persist. This recommendation by no means replaces other measurements in the follow-up of ADH1 patients such as 24 h calcium excretion or serum creatinine, but it provides another tool to guide the physician. Further studies in larger groups of patients with various *CASR* mutations are needed to examine the effect of implementing our paradigm for the prevention of both hypocalcemia-related neurological symptoms and hypercalciuria with subsequent renal impairment in patients with ADH1.

## Figures and Tables

**Table 1.** Clinical and biochemical characteristics of the 7 affected members

Subject No.	Age*, years / Gender	Serum** calcium, mg/dL	Serum phosphate, mg/dL	Serum magnesium, mg/dL	Serum PTH, pg/mL	Urine Ca/Cr, mg/mg	Serum^#^ creatinine, mg/dL	eGFR^#^ mL/min/1.73 m^2^	Seizures	Nephro calcinosis (age, Y)^¶^	Nephro lithiasis (age, Y)^¶^	Lens opacity (age, Y)^¶^
I-1	39 F	5.5	5.0	1.6	8.0	na	1.35	48	−	−	−	+(27)
II-1	14 F	6.2	6.9	na	9.0	0.24	1.5	48	−	+(24)	+(26.1)	−
II-2	0.2 F	7.6	10.0	na	na	na	0.6	126	−	−	−	+(2.3)
II-3	0 F	7.0	6.7	1.6	10.0	na	0.8	105	−	+(8)	−	−
III-2	1.6 F	8.6	6.1	na	<4	0.44	0.4	160	−	+(2.7)	+(2.8)	−
III-3	0 M	7.7	6.9	na	<4	na	0.4	180	+	−	−	−
III-4	0 M	7.5	9.6	na	4.0	0.89	0.2	223	+	−	−	−

Normal ranges in serum: calcium: 8.8–10.8 mg/dL; phosphate 3.3–5.4 mg/dL; magnesium 1.7–2.1 mg/dL; PTH 6.5–36.8 pg/mL; creatinine 0.6–1.2 mg/dL. F, female; M, male; eGFR, estimated glomerular filtration rate (Levey et al. [14]); na, not available.

* At the time of initial biochemical studies.

** Total serum calcium.

# Last available measurement.

¶ Age at diagnosis.

**Table 2.** Age-adjusted highest levels of serum calcium associated with calcium excretion in the upper normal range for patients with ADH1

Age range, years	Urinary Ca/Cr ratio, mg/mg; 95th percentile*	Serum calcium, mg/dL
1st year	0.81	8.8
2nd year	0.56	8.1
3rd year	0.50	7.9
3–5	0.41	7.6
5–7	0.30	7.3
7 to adult	0.22–0.25	7.1

* Based on Matos et al. [16] for the age range of birth to 7 years and both Matos et al. [16] and Sargent et al. [15] for the age range of 7 years to adulthood.

## Data Availability

The data that support the findings of this study are not publicly available due to information that could compromise the privacy of research participants but are available from the corresponding author (A.Z.) upon reasonable request.
